# Activating health behaviors: how does digital infrastructure improve the physical health of middle-aged and older adults? Evidence from the ‘Broadband China’ pilot policy

**DOI:** 10.1080/16549716.2025.2568779

**Published:** 2025-10-09

**Authors:** Zhiying Li, Yunhui Wang, Longhua Zheng, Hong Xu

**Affiliations:** a School of Government, University of Chinese Academy of Social Sciences, Beijing, China; b School of Public Administration, Sichuan University, Chengdu, China; c English Department, Jining Shiqiao Middle School, Jining, China; d School of Public Administration and Policy, Shandong University of Finance and Economics, Jinan, China

**Keywords:** digital divide, public health intervention, health behavior, health inequality, quasi-experimental design

## Abstract

**Background:**

The rapid development of digital infrastructure has the potential to affect public health, particularly among middle-aged and older adults. However, the causal relationship between digital infrastructure and physical health remains underexplored, especially in non-Western contexts.

**Objectives:**

This study investigates the causal effect of digital infrastructure development on the physical health of middle-aged and older adults in China, using panel data from the China Health and Retirement Longitudinal Study (CHARLS, 2011–2020) and a DID approach based on the Broadband China pilot policy.

**Methods:**

We construct a quasi-natural experiment using the Broadband China policy to examine its impact on physical health. The DID model analyzes health outcomes before and after the policy, focusing on physical health and health-related expenditures. Subgroup analyses are conducted by age and education.

**Results:**

The findings show that (1) Digital infrastructure is associated with modest improvements in physical health, reflected in a small reduction in activity limitations; (2) Health behaviors – such as slightly increased physical exercise and marginally higher health-related spending – partially mediate these effects; (3) The relative benefits are more pronounced among older adults and those with lower education; (4) Digital infrastructure may help partially reduce health inequalities, particularly among individuals with poorer baseline health.

**Conclusion:**

Digital infrastructure is associated with modest improvements in physical health among middle-aged and older adults, partly through promoting healthier behaviors. It may contribute to reducing health disparities and offer policy implications for enhancing health equity, particularly among vulnerable populations.

## Paper Context

**● Main findings**Digital infrastructure development is associated with modest improvements in the physical health of middle-aged and older adults, partly through promoting healthier behaviors such as physical exercise and increased health-related spending, and may help reduce health inequalities.

**● Added knowledge** This study contributes to digital health research by highlighting behavioral pathways through which macro-level infrastructure interventions may affect population health.

**● Global health impact for policy and action** Investing in digital infrastructure may support healthier aging and help mitigate behavioral health disparities in aging societies, complementing other individual- and community-level interventions.

## Background

Population aging has emerged as a critical global challenge. According to the United Nations’ World Population Prospects 2024, the number of people aged 65 and over is projected to reach 2.2 billion by the late 2070s, surpassing the population under 18 for the first time. In China, individuals aged 65 and above accounted for 15.6% of the population in 2024, and this share is expected to rise in the coming decades. This demographic shift places substantial pressure on healthcare and social security systems and on broader economic development, because middle-aged and older adults are generally more susceptible to chronic illness and functional decline. Consequently, promoting healthy aging has become a national priority. These demographic challenges highlight the urgency of exploring innovative solutions, among which digital infrastructure plays a pivotal role.

At the same time, the rapid expansion of digital infrastructure is changing access to information and services. Evidence shows that Internet expansion can improve access to healthcare services, facilitate telemedicine, and support health-related decision-making [1–4]. However, previous studies have given limited attention to infrastructure-level effects – that is, how the rollout of broadband itself influences health via behavioral pathways – and to whether these effects differ across social groups.

In particular, it remains unclear whether improved access to health information and reduced service costs translate into sustained health behaviors. Individuals with lower education or digital literacy may be less able to benefit from digital expansion, potentially exacerbating health inequalities [5–7]. Addressing these gaps is essential for designing inclusive digital health policies.

This study leverages the phased rollout of the Broadband China pilot as a quasi-natural experiment and utilizes CHARLS data (2011–2020) to examine how digital infrastructure development influences the health of middle-aged and older adults through behavioral mechanisms. Our contributions are threefold. First, we shift the focus from individual Internet use to the structural role of infrastructure, highlighting mechanisms that have been underexplored. Second, we identify health behaviors as a mediating channel, thereby revealing micro-foundations of how infrastructure shapes health outcomes. Third, we examine heterogeneity across social groups to inform stratified and equitable health policy design.

## Policy background and theoretical framework

### Policy background

Launched by the State Council in August 2013, the Broadband China strategy marked the formal recognition of broadband networks as a component of the national strategic infrastructure. The policy outlined two developmental phases: the first (by 2015) aimed to expand broadband coverage and user access; the second (by 2020) sought to achieve universal coverage in urban and rural areas, popularize mobile internet, and promote broadband integration across sectors.

To implement the strategy, the Ministry of Industry and Information Technology and the National Development and Reform Commission jointly initiated three rounds of pilot city selection between 2014 and 2016, ultimately designating 120 cities. The selection involved a dual-review process – provincial pre-screening and national evaluation – and prioritized cities with adequate broadband foundations and demonstrable potential for improvement and regional leadership. Notably, despite favoring better-resourced regions, not all applicant cities were selected. For example, only 3 of the 14 cities from Sichuan Province were approved in 2014, reflecting a selection rate of less than 25%.

The implementation of this policy exhibits a typical staggered and gradual pattern, with variations in timing across cities in both spatial and temporal dimensions. This staggered rollout generates plausibly exogenous variation in treatment status, as cities entered the program at different times for administrative and technical reasons rather than local health trends. Such a variation enables the construction of comparable treatment and control groups over time, thereby providing a credible identification strategy. Therefore, the rollout of the ‘Broadband China’ initiative provides a suitable quasi-natural experiment for a staggered difference-in-differences design, facilitating the identification of the causal effects of digital infrastructure development on social outcomes.

From a health perspective, broadband expansion improves access to health information and services, particularly in underserved regions. Enhanced connectivity facilitates the dissemination of health information and supports the development of telemedicine, online consultation, and chronic disease monitoring, thereby addressing structural disadvantages in healthcare access among middle-aged and older adults. Accordingly, this study treats the Broadband China pilot as an exogenous policy shock and exploits the variation in city-level implementation timing between 2014 and 2016 to construct a staggered DID model, identifying the net effect of digital infrastructure on physical health.

### Theoretical framework

Resocialization theory posits that individuals must continually adapt to changing social environments and acquire new skills across the life course to maintain social integration [8]. For middle-aged and older adults, declining physical function and shifting social roles can hinder effective health management. Digital infrastructure provides tools that facilitate timely access to health knowledge, support self-management strategies, and enhance connections with healthcare resources, enabling individuals to better respond to evolving health needs [9]. Based on this reasoning, we propose:

H1: Digital infrastructure development enhances the physical health of middle-aged and older adults.

Network gain theory suggests that the Internet improves access to accurate and relevant information, thereby supporting more informed decision-making [10]. In the context of health, digital infrastructure can efficiently disseminate medical knowledge, improve awareness of preventive measures, and facilitate disease management [11]. These benefits are particularly relevant for chronic disease control and navigating healthcare services, helping individuals overcome information asymmetries [12].

Activity theory emphasizes that maintaining active social and physical engagement contributes to better health outcomes in later life [13]. Regular physical activity improves cardiovascular function, reduces chronic disease risk, and slows physiological decline [14–17]. However, traditional exercise interventions face barriers for many middle-aged and older adults, including limited knowledge, restricted access, and insufficient self-management capacity [18]. Digital infrastructure can address these challenges: smart health devices allow real-time monitoring and feedback-based exercise adjustments, while digital platforms provide personalized guidance and motivation, fostering sustained physical activity, thereby promoting physical health among middle-aged and older adults [19,20]. Based on this mechanism, we propose:

H2a: Digital infrastructure development promotes physical health by increasing physical exercise among middle-aged and older adults.

Quality of life is shaped not only by physical health but also by social relationships, economic resources, and psychological well-being [21]. Consumer behavior theory highlights that decision-making is shaped by environmental and contextual factors [22]. With digital technology, access to health-related information and online services transforms consumption patterns and enables more effective health-related expenditures [23–25]. By lowering barriers to purchasing health products and expanding knowledge about effective interventions, digital infrastructure enables more informed health-related expenditures, which in turn contributes to improved physical health. Drawing on this behavioral channel, we propose:

H2b: Digital infrastructure development promotes physical health by increasing health-related expenditures among middle-aged and older adults.

Taken together, Activity Theory and Consumer Behavior Theory suggest that the influence of digital infrastructure on physical health is not direct, but rather operates through the channel of observable health behaviors. We therefore posit two distinct behavioral pathways: one through the promotion of physical activity (H2a), and the other through the facilitation of more informed health-related expenditures (H2b). By influencing these key health behaviors, digital infrastructure contributes to improvements in the physical health of middle-aged and older adults.

Finally, digital infrastructure can help to reduce health disparities. Unequal access to health information and medical resources is a major source of inequality, with lower-educated or low-income individuals often disadvantaged [26–28]. Digital tools – such as health apps, online education platforms, and telemedicine services – enhance access to knowledge, optimize resource distribution, and lower financial barriers, thereby mitigating health inequalities among middle-aged and older adults. We formalize this as:

H3: Digital infrastructure development reduces physical health inequalities within the middle-aged and older adult group.

**Figure 1.**
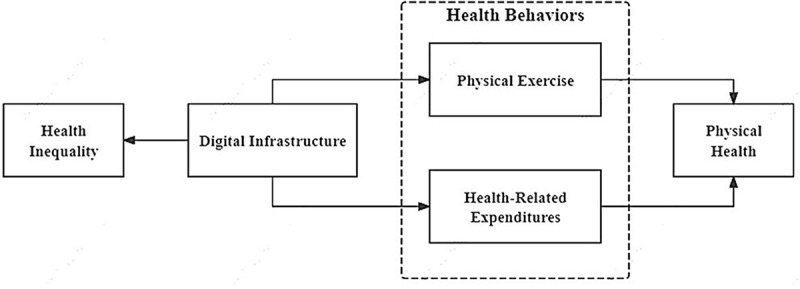
Research framework.

In conclusion, the research framework for this study is presented in Figure 1

## Methods

### Data sources

This study utilizes data from the China Health and Retirement Longitudinal Study (CHARLS), a nationally representative survey administered by Peking University that covers 150 counties and districts across China. The dataset is built upon a rigorous sampling framework and high-quality face-to-face interviews, collecting comprehensive information on health status, economic conditions, social security, and family structure. Widely used in health economics and public policy research, CHARLS provides a solid empirical basis for analyzing the health effects of digital infrastructure development among middle-aged and older adults.

CHARLS employs a longitudinal design that allows for the construction of panel data, thereby controlling for unobservable individual characteristics and time trends. This makes it suitable for applying a difference-in-differences (DID) approach to identify the causal impact of the Broadband China pilot policy. This study uses five survey waves—2011, 2013, 2015, 2018, and 2020—and limits the sample to individuals aged 45 and above. After excluding cases with missing key variables (approximately 8.6% of the sample), the final analytic sample consists of 51,866 observations. The excluded individuals showed no systematic differences from those retained, indicating that the missing data are mostly random and unlikely to bias the results.

### Variable definitions

The key explanatory variable is digital infrastructure development, measured using the implementation status of the Broadband China strategy, which was launched by the State Council in 2013 to expand broadband coverage, improve service quality, and promote digital applications. The policy’s phased rollout introduced regional variation suitable for quasi-experimental analysis. Following Peng et al. [29], He et al. [30], and Jia and Li [31], we use a binary indicator for the pilot policy: if individual *i* resides in city *c* and year *t* corresponds to a period after the city entered the pilot, then did = 1; otherwise, did = 0.

The dependent variable is the physical health of middle-aged and older adults, measured using the KATZ Index of Independence in Activities of Daily Living [32]. This includes six basic functional abilities: eating, dressing, bathing, toileting, walking, and continence. For each item, a value of 1 indicates difficulty or inability, and 0 indicates no difficulty. These are summed to construct a continuous variable ranging from 0 to 6, where lower scores represent better health status. Individuals with missing responses on any of the six ADL items were excluded from the analysis. To assess the robustness of our results to different coding strategies, we also conducted sensitivity analyses by categorizing ADL into three groups (0, 1–2, 3–6) representing low, moderate, and high limitations, respectively. The results were consistent in both direction and significance, indicating that the main findings are robust to alternative ADL codings.

The mediating variables in this study include two key health behaviors: physical exercise and health-related expenditures. Physical exercise is coded as 1 if the respondent engages in at least 10 minutes per week of light, moderate, or vigorous physical activity; otherwise, it is coded as 0. This variable captures whether an individual participates in any form of regular physical activity, thereby reflecting potential changes in exercise behavior resulting from improvements in the digital infrastructure. Health-related expenditures are based on household spending over the past year on fitness, supplements, and health equipment. We use per capita household expenditures, adding one before taking the natural logarithm to accommodate zero values, reduce skewness, and enhance the interpretability and robustness of the estimates. Health-related expenditures provide an objective measure of household investment and behavioral adjustment toward maintaining health, reflecting potential changes in health investment associated with digital infrastructure improvements.

We also control for additional factors that may influence the health of middle-aged and older adults, including individual-level characteristics (residence, gender, age, education, ethnicity, pension coverage, health insurance status, chronic disease conditions) and household-level characteristics (marital status, household size). To partly account for the potential effects of economic development and environmental conditions on both infrastructure rollout and health outcomes, we include two city-level macro controls: per capita GDP and industrial sulfur dioxide (SO₂) emissions, with data drawn from the China City Statistical Yearbook. City and year-fixed effects are also incorporated, and standard errors are clustered at the city level to mitigate the influence of regional economic conditions and unobserved heterogeneity. Descriptive statistics are presented in Table 1

**Table 1.** Variable definitions and descriptive statistics.

Variable Type	Variable Name	Variable Definition and Assignment	Obs	Mean	SD	Min	Max
Dependent Variable	physical health	A continuous variable constructed based on the ADL scale (Lower score indicates better health status)	51,866	0.282	0.770	0	6
Core Explanatory Variable	did	‘Broadband China’ pilot policy × year	51,866	0.223	0.417	0	1
Control Variables	residence	Respondent’s residence (Urban = 0, Rural = 1)	51,866	0.630	0.483	0	1
	gender	Respondent’s gender (Female = 0, Male = 1)	51,866	0.494	0.500	0	1
	age	Respondent’s actual age at the time of the survey	51,866	61.165	9.166	46	120
	education	Respondent’s education (Below Primary = 1, Primary = 2, Secondary = 3, High School and Above= 4)	51,866	2.045	1.059	1	4
	ethnicity	Ethnicity of Respondents (Non-Han = 0, Han = 1)	51,866	0.952	0.214	0	1
	pension	Respondent’s pension insurance status (No pension insurance = 0, Any pension insurance = 1)	51,866	0.588	0.492	0	1
	health insurance	Respondent’s health insurance status (No health insurance = 0, Any health insurance = 1)	51,866	0.959	0.199	0	1
	chronic disease	Whether the respondent has chronic diseases (No = 0, Yes = 1)	51,866	0.761	0.426	0	1
	marital status	Respondent’s marital status (Not married = 0, Married = 1)	51,866	0.881	0.324	0	1
	household size	Number of family members of the respondent	51,866	3.167	1.632	1	16
	per capita GDP	ln(city-level per capita gross domestic product +1)	51,866	10.646	0.576	8.842	12.153
	industrial sulfur dioxide emissions	ln(city-level total industrial SO₂ emissions +1)	51,866	9.898	1.307	6.052	13.183
Mechanism variables	physical exercise	Respondent’s answer to the question, ‘Do you engage in at least 10 minutes of physical activity per week?’ (No = 0, Yes = 1)	35,878	0.916	0.277	0	1
	health-related expenditures	ln (Past year’s health-related expenditures +1)	51,088	0.372	1.459	0	10.127

### Model specification

In empirical social science research, the DID method is widely used to identify the causal effects of policy interventions by treating them as exogenous shocks. The Broadband China pilot began in 2014 and was implemented in waves across different cities, introducing both temporal variation within cities and cross-sectional variation between pilot and non-pilot cities. This phased rollout lends itself to a staggered DID design. Accordingly, we treat the policy as a multi-period, multi-group quasi-natural experiment and estimate the net effect of the policy on physical health by comparing differences in health outcomes before and after implementation between treated and untreated cities. The baseline regression model is specified as follows:

(1) $$\mathrm{Healt}h_{\mathrm{ict}}=\alpha_{0}+\beta_{1}\mathrm{DI}D_{\mathrm{ict}}+\beta_{2}\mathrm{Control}s_{\mathrm{ict}}+\gamma_{c}+\delta_{t}+\varepsilon_{\mathrm{ict}}$$

where subscripts *i*, *c*, and *t* denote individual, city, and year, respectively. $\mathrm{Healt}h_{\mathrm{ict}}$ is the dependent variable measuring the physical health of individual *i* in city *c* at time *t*. $\mathrm{DI}D_{\mathrm{ict}}$ is the treatment indicator, defined as the interaction between the post-policy period and pilot city status. $\mathrm{Control}s_{\mathrm{ict}}$ is a vector of control variables at the individual and household levels. $\delta_{t}$ and $\gamma_{c}$ represent year and city-fixed effects, respectively. $\varepsilon_{\mathrm{ict}}$ is the error term. Robust standard errors are clustered at the city level.

A possible concern is that the designation of pilot cities under the Broadband China initiative was not entirely random, which may introduce policy selection bias. To mitigate this issue, we control for a broad set of individual-, household-, and city-level characteristics, and include city- and year-fixed effects to absorb time-invariant heterogeneity and common shocks. In addition, we conducted a series of robustness checks – including parallel trend and placebo tests, propensity score matching combined with DID (PSM-DID), substitution of the dependent variable, sample period adjustment, and controls for concurrent policies. These strategies complement each other: covariates and fixed effects account for observable and unobservable confounders, while PSM-DID improves baseline comparability between treated and untreated cities, and placebo and falsification tests guard against spurious correlations. The consistency of results across these specifications provides reassurance that the estimated effects are not primarily attributable to non-random policy assignment, thereby strengthening the causal interpretation.

Drawing on the research of Li et al. [33] and Turguttopbaş [34], this study adopts the Kakwani Relative Deprivation Index to measure health inequality. Let Y represent a reference group with a sample size of n, and let the middle-aged and older adults’ health levels be ranked, yielding an overall health distribution vector y= ($y_{1},y_{2},y_{3},y_{n-1},y_{n}$), where $y_{1}\leq y_{2}\leq y_{3}\leq y_{n-1}\leq y_{n}$. Therefore, the relative deprivation index $\mathrm{RD}\left(y_{j},y_{i}\right)$ for individual i’s health compared to individual j is defined as:

(2) $$\mathrm{RD}\left(y_{j},y_{i}\right)=\left\{\begin{array}{c} y_{j}-y_{i}\;\mathrm{if}\;y_{j}\;>\;y_{i}\\ \;\;\;\;\;\;0\;\mathrm{if}\;y_{j}\leq y_{i} \end{array}\right.$$

Building on Equation (2), the average health relative deprivation index $\mathrm{RD}\left(y,y_{i}\right)$ for individual i is calculated as:

(3) $$\mathrm{RD}\left(y,y_{i}\right)=\frac{1}{n\mu_{Y}}\left(n_{y_{i}}^{+}\times \mu_{y_{i}}^{+}+n_{y_{i}}^{+}\times y_{i}\right)$$

Where $\mu_{y}$ is the mean health of all samples in the reference group Y, $n_{y_{i}}^{+}$ is the number of middle-aged and older adults in the reference group Y whose health exceeds $y_{i}$, and $\mu_{y_{i}}^{+}$ is the mean health of middle-aged and older adults in the reference group Y whose health exceeds the health level of $y_{i}$.

## Results

### Baseline regression

Table 2 reports the baseline regression results on the impact of digital infrastructure development on the physical health of middle-aged and older adults. Model 1 includes only the core explanatory variable, with the interaction term being negative and statistically significant. Model 2 adds controls for individual, household, and city-level characteristics, and the results remain robust. Model 3 further incorporates time and regional-fixed effects, with the interaction term estimated at −0.0573, significant at the 1% level, indicating that digital infrastructure development significantly improves the health status of middle-aged and older adults. In practical terms, this coefficient indicates that the policy reduces ADL scores by approximately 0.0573 points on the 0–6 scale, corresponding to about a 1% reduction in functional limitations, representing a meaningful enhancement in overall health for the middle-aged and older population.

**Table 2.** Baseline regression results.

Variable	Model1	Model2	Model3
did	−0.0344***	−0.0303***	−0.0573***
	(0.0081)	(0.0086)	(0.0195)
gender		−0.1046***	−0.1074***
		(0.0069)	(0.0124)
residence		−0.0663***	−0.0739***
		(0.0107)	(0.0182)
age		0.0806***	0.0662***
		(0.0072)	(0.0162)
education		0.0011	0.0051*
		(0.0021)	(0.0027)
ethnicity		0.0137***	0.0141***
		(0.0005)	(0.0010)
pension		−0.0389***	−0.0373***
		(0.0034)	(0.0049)
health insurance		−0.0495***	−0.0642**
		(0.0153)	(0.0303)
chronic disease		0.0180**	0.0122
		(0.0082)	(0.0108)
marital status		−0.0608***	−0.0525***
		(0.0165)	(0.0198)
household size		0.1924***	0.1791***
		(0.0078)	(0.0098)
per capita GDP		−0.0651***	0.0017
		(0.0063)	(0.0336)
industrial sulfur dioxide emissions		−0.0162***	0.0042
		(0.0026)	(0.0096)
_cons	0.2901***	0.3851***	−0.5210
	(0.0038)	(0.0800)	(0.3770)
time-fixed effects	No	No	Yes
regional-fixed effects	No	No	Yes
*N*	51,866	51,866	51,866
adj. *R*^2^	0.0003	0.0738	0.0873

Standard errors in parentheses.

*p < 0.1, **p < 0.05, ***p < 0.01.

### Robustness checks

To ensure the reliability of the findings, we conducted a series of robustness checks, including a parallel trend test, placebo test, PSM-DID, substitution of the dependent variable, sample period adjustment, and controlling for concurrent policy effects.

**Figure 2.**
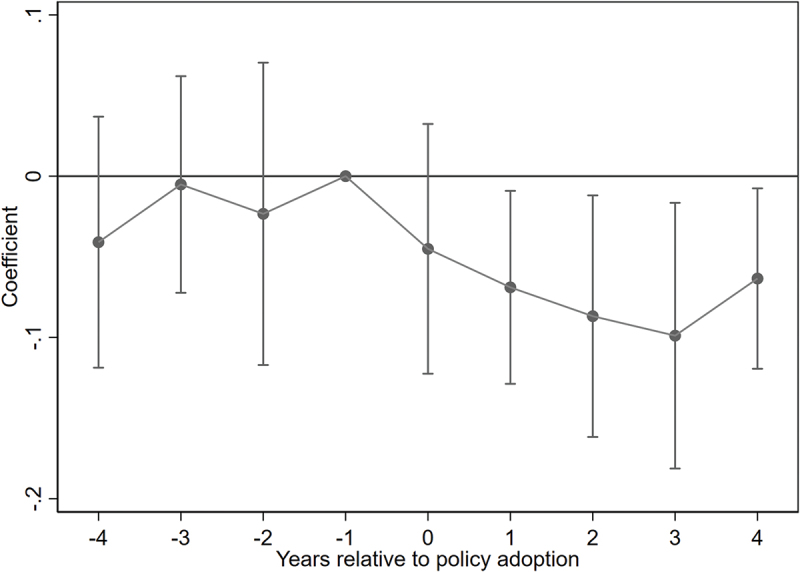
Parallel trend test.

Parallel Trend Test. The DID approach relies on the parallel trend assumption, which posits that, prior to policy implementation, the treatment and control groups should exhibit similar health trends. We conducted an event-study analysis using the wave prior to the policy rollout as the reference period. Figure 2 reports the estimated coefficients and their 95% confidence intervals. The results show that the pre-treatment coefficients fluctuate around zero and the confidence intervals include zero, supporting the validity of the parallel trend assumption. Post-implementation coefficients are significantly negative, indicating that the Broadband China pilot effectively improved health among middle-aged and older adults. These effects are not driven by pre-existing trends, confirming the robustness of the results.

**Figure 3.**
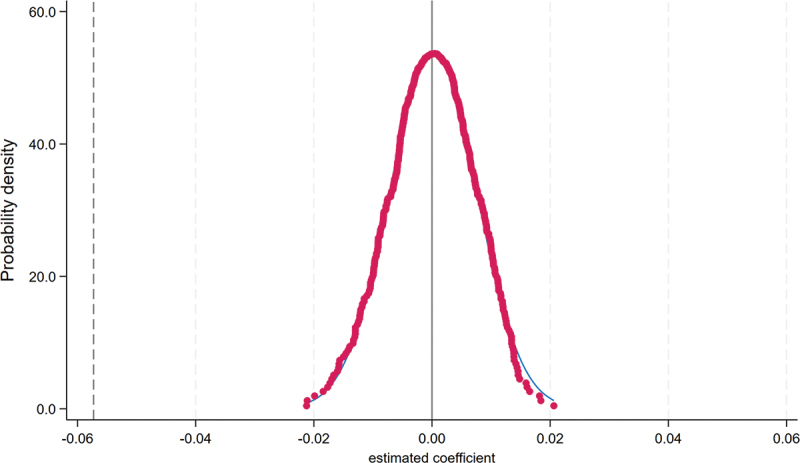
Placebo test.

Placebo Test. To further assess the robustness of our identification strategy, we conducted a placebo test using a non-parametric permutation method. Specifically, we kept the original sample structure intact but randomly shuffled the treatment indicator across observations. For each of the 500 iterations, we re-estimated the baseline regression model with the permuted treatment variable, including all original covariates and fixed effects. The resulting distribution of placebo coefficients is plotted in Figure 3. These estimates are centered around zero and are largely statistically insignificant, indicating that the main treatment effect we find is unlikely due to chance, model misspecification, or sample selection bias. This placebo test thus reinforces the credibility of our causal inference.

PSM-DID. To account for potential selection bias between pilot and non-pilot cities, we employed the PSM-DID method. Using nearest-neighbor matching (1:4) and kernel matching, we selected control units with similar socioeconomic characteristics from non-pilot areas. DID regressions were then conducted on the matched sample. As shown in Table 3, the estimated policy effects remain significantly negative, reinforcing the conclusion that digital infrastructure development positively influences health outcomes among middle-aged and older adults.

**Table 3.** Robustness checks.

Variable	Model1	Model2	Model3	Model4	Model5
	PSM-DID		Substitution of the Dependent Variable	Sample Period Adjustment	Controlling for Concurrent Policy Effects
did	−0.0512**	−0.0580***	−0.2992***	−0.0682***	−0.0555***
	(0.0210)	(0.0197)	(0.1019)	(0.0201)	(0.0196)
Smart City pilot					−0.0469*
					(0.0250)
control variables	Yes	Yes	Yes	Yes	Yes
_cons	−0.3890	−0.5342	−0.5379	−0.2841	−0.5479
	(0.4054)	(0.3802)	(2.7313)	(0.5579)	(0.3652)
time-fixed effects	Yes	Yes	Yes	Yes	Yes
regional-fixed effects	Yes	Yes	Yes	Yes	Yes
*N*	42,567	51,783	51,866	39,914	51,866
adj. *R*^2^	0.0847	0.0872	0.1655	0.0870	0.0874

Standard errors in parentheses.

*p < 0.1, **p < 0.05, ***p < 0.01.

Substitution of the Dependent Variable. Given that physical pain is closely associated with poor health status [35], we conducted a robustness check by substituting the original ADL-based health measure with a pain index. This index is constructed by summing the number of reported pain sites, ranging from 0 to 15, with higher values indicating greater pain. Model 3 in Table 3 shows that even with the alternative measure, digital infrastructure development remains significantly associated with improved health, further validating the robustness of our findings.

Sample Period Adjustment. The COVID-19 pandemic significantly disrupted global health systems and individual health status, potentially confounding the policy effects. To address this, we excluded data from 2020 and re-estimated the baseline model. As reported in Model 4 of Table 3, the coefficient of the Broadband China pilot remains significant at the 1% level (−0.0682), reaffirming the positive health impact of digital infrastructure development.

Controlling for Concurrent Policy Effects. To rule out potential confounding from other contemporaneous policies, we controlled for the Smart City pilot program, which overlaps in both timing and geographic coverage with Broadband China and also targets improvements in digital infrastructure and public services. We included a binary variable indicating whether the respondent’s city was a Smart City pilot in that year. Model 5 of Table 3 shows that the inclusion of this control does not materially alter the estimated effect, which remains negative and statistically significant. This confirms that the health benefits attributed to Broadband China are not driven by other digitalization-related policies.

### Mechanism testing

To examine the mechanisms through which digital infrastructure development affects physical health, we introduced physical exercise and health-related expenditures as mediating variables. Table 4 reports the results of mediation analysis for these two channels. The findings indicate that digital infrastructure development significantly promotes physical exercise participation and increases health-related expenditures among middle-aged and older adults, both of which are associated with improved health outcomes.

**Table 4.** Mechanism test.

Variable	Model1	Model2	Model3	Model4
	Physical Exercise	Health	Health-Related Expenditures	Health
did	0.0203*	−0.0551**	0.0911**	−0.0578***
	(0.0106)	(0.0233)	(0.0394)	(0.0196)
physical exercise		−0.2626***		
		(0.0252)		
health-related expenditures				−0.0054***
				(0.0020)
control variables	Yes	Yes	Yes	Yes
_cons	0.9470***	−0.5226	0.0785	−0.5499
	(0.1736)	(0.4176)	(0.6722)	(0.3778)
time-fixed effects	Yes	Yes	Yes	Yes
regional-fixed effects	Yes	Yes	Yes	Yes
*N*	35,878	35,878	51,088	51,088
adj. *R*^2^	0.0397	0.0986	0.0520	0.0874

Standard errors in parentheses.

*p < 0.1, **p < 0.05, ***p < 0.01.

We further tested the significance of the mediating effects using the Bootstrap method in Table 5. If the Bootstrap confidence intervals (CIs) for the indirect effects exclude zero, the mediation is considered significant. Table 5 shows that the indirect effect of physical exercise (95% CI: −0.0091, −0.0016) and health-related expenditures (95% CI: −0.0009, −0.0001) are both statistically significant. These findings align with the regression results and confirm that digital infrastructure development improves physical health by facilitating healthier behaviors.

**Table 5.** Mediation effect test based on the Bootstrap method.

Mediator Variable	Effect Type	Coefficient	SE	95% conf. interval	
physical exercise	indirect effect	−0.0053	0.0019	−0.0091	−0.0016
	direct effect	−0.0551	0.0176	−0.0895	−0.0207
health-related expenditures	indirect effect	−0.0005	0.0002	−0.0009	−0.0001
	direct effect	−0.0578	0.0143	−0.0859	−0.0297

### Heterogeneity analysis

Given variation in socioeconomic status, health conditions, and digital literacy, the effects of digital infrastructure development may differ across population subgroups. Table 6 presents the results from the subgroup analyses. The policy has a more pronounced effect on older adults (Model 2) and individuals with lower education levels (Model 3), with coefficients of −0.0764 (*p* < 0.05) and −0.0590 (*p* < 0.01), respectively. In contrast, the effects for middle-aged individuals (Model 1) and those with higher education (Model 4) are weaker and less stable. These results suggest that digital infrastructure development has heterogeneous effects and may offer greater health benefits for older and less-educated populations, highlighting its potential in addressing health inequality.

**Table 6.** Heterogeneity analysis.

Variable	Model1	Model2	Model3	Model4
	Age < 60	Age ≥ 60	Low Education	High Education
did	−0.0250*	−0.0764**	−0.0590***	−0.0421
	(0.0149)	(0.0310)	(0.0210)	(0.0276)
control variables	Yes	Yes	Yes	Yes
_cons	0.1826	−1.1053*	−0.4234	−1.0127**
	(0.2993)	(0.6086)	(0.4057)	(0.4917)
time-fixed effects	Yes	Yes	Yes	Yes
regional-fixed effects	Yes	Yes	Yes	Yes
*N*	23,811	28,055	45,512	6354
adj. *R*^2^	0.0475	0.0778	0.0863	0.0503

Standard errors in parentheses.

*p < 0.1, **p < 0.05, ***p < 0.01.

### Further analysis

Table 7 presents the estimation results of the impact of digital infrastructure development on health inequality among older adults. Using the Kakwani relative deprivation index, the health variable was reverse-coded so that higher scores indicate better health, allowing the index to capture relative health disadvantage within each city-year group. Model 1 excludes control variables, Model 2 adds individual-, household-, and city-level covariates, and Model 3 further incorporates time and regional-fixed effects. In Model 3, the coefficient of the core explanatory variable (did) is −0.0083 (*p* < 0.01), indicating that digital infrastructure development significantly reduces health inequality among middle-aged and older adults. In substantive terms, this corresponds to an average reduction of approximately 0.83% in relative health disadvantage, reflecting a meaningful improvement in health equity. These results provide further evidence that digital infrastructure plays an important role in mitigating health disparities in later life.

**Table 7.** The impact of digital infrastructure development on health inequality.

Variable	Model1	Model2	Model3
did	−0.0050***	−0.0049***	−0.0083***
	(0.0012)	(0.0013)	(0.0026)
gender		−0.0149***	−0.0152***
		(0.0010)	(0.0018)
residence		−0.0107***	−0.0117***
		(0.0016)	(0.0028)
age		0.0111***	0.0094***
		(0.0011)	(0.0024)
education		0.0003	0.0008*
		(0.0003)	(0.0004)
ethnicity		0.0020***	0.0021***
		(0.0001)	(0.0001)
pension		−0.0057***	−0.0055***
		(0.0005)	(0.0007)
health insurance		−0.0084***	−0.0101**
		(0.0023)	(0.0049)
chronic disease		0.0028**	0.0022
		(0.0012)	(0.0016)
marital status		−0.0091***	−0.0081***
		(0.0025)	(0.0030)
household size		0.0276***	0.0260***
		(0.0012)	(0.0014)
per capita GDP		−0.0079***	0.0002
		(0.0010)	(0.0045)
industrial sulfur dioxide emissions		−0.0019***	0.0006
		(0.0004)	(0.0014)
_cons	0.0414***	0.0351***	−0.0771
	(0.0006)	(0.0122)	(0.0507)
time-fixed effects	No	No	Yes
regional-fixed effects	No	No	Yes
*N*	51,866	51,866	51,866
adj. *R*^2^	0.0003	0.0671	0.0762

Standard errors in parentheses.

*p < 0.1, **p < 0.05, ***p < 0.01.

## Discussion

This study draws on panel data from CHARLS from 2011 to 2020 and employs a DID approach, leveraging the quasi-natural experiment of the Broadband China pilot policy, to evaluate the impact of digital infrastructure development on the physical health of middle-aged and older adults. The analysis further explores the underlying mechanisms, heterogeneity across subpopulations, and implications for health inequality. The main findings are as follows:

First, digital infrastructure development is associated with modest improvements in the physical health of middle-aged and older adults. While the estimated reduction in ADL limitations is modest in magnitude, even small improvements in physical functioning can be meaningful at the population level, particularly among older adults who are at risk of functional decline. These small individual gains may accumulate across communities, reducing the burden of disability and contributing to healthier aging trajectories.

This finding, highlighting that infrastructure-level interventions, can influence population health outcomes, although the effects are incremental. In contrast to existing studies that focus on the direct health effects of digital usage [36,37], this study adopts a macro-infrastructure perspective, identifying broad spillover effects. It enhances the theoretical understanding of how digital technologies affect health outcomes and provides empirical evidence that may inform policies aiming to optimize public health benefits through infrastructure investment. These findings contribute to both digital health and active aging theories. From a digital health perspective, individual health is not solely determined by medical behaviors but is also shaped by access to information, distribution of health resources, and technological infrastructure. Digital infrastructure may enhance the ability of middle-aged and older adults to access health services and information, which may broaden the factors influencing health. From the standpoint of aging theory, environmental adaptability is central to healthy aging [38]. Infrastructure development is associated with indicators of improved health among aging populations, suggesting a potential role in supporting functional maintenance and aging in place.

Thus, this study not only empirically verifies the health benefits of digital infrastructure but also highlights the structural determinants of health in later life, advancing the research frontier on technological empowerment for aging well-being. Although the estimated effects on physical functioning are modest, even small improvements can accumulate across populations, contributing to healthier aging. These findings highlight the potential of digital infrastructure to support functional maintenance while suggesting that additional interventions at the individual and community level may further enhance health outcomes.

Second, digital infrastructure development promotes better health behaviors among middle-aged and older adults, thereby enhancing their health outcomes. Previous studies have shown that artificial intelligence [39], access to health information [40], and medical service availability [41] significantly influence health decisions. However, prior research has focused mainly on the effects of individual-level internet usage, with limited attention to how systemic factors like infrastructure shape the evolution of health behavior. Some scholars have emphasized the role of health knowledge [42] and social class [43] in mediating the impact of digital technologies on health, but few have systematically assessed the comprehensive effects of infrastructure development. This study provides empirical evidence that digital infrastructure is associated with small increases in physical exercise and health-related expenditures, suggesting partial behavioral pathways that contribute to health improvements. Three mechanisms may explain these effects: First, digital infrastructure reduces the cost of accessing health information, enabling individuals to make more informed decisions about disease prevention, nutrition, and exercise. Second, digital technologies enhance the social environment [44], facilitating peer-based health communication and reinforcing positive behaviors through group dynamics. Third, wearable devices and digital health management tools offer real-time feedback and motivational incentives, improving behavioral adherence and sustainability [45]. These findings underscore the systemic role of digital infrastructure in health promotion and expand the scope of information technology’s influence on health behaviors, providing insights that could inform public health policy.

Third, the health effects of digital infrastructure development vary significantly across demographic subgroups. Prior research indicates that age [46], living environment [47], and education level [48] affect individuals’ capacity to adopt digital technologies and engage in health-related behaviors. However, much of this literature focuses on isolated factors and lacks a comprehensive analysis of how structural conditions mediate health outcomes. This study fills that gap by examining how age and human capital shape the heterogeneous impacts of infrastructure. The findings show that the positive health effects of digital infrastructure are more pronounced among older adults and individuals with lower educational attainment, while the effects are weaker among middle-aged and highly educated groups. This heterogeneity may stem from differences in health needs, resource access, and adaptation capacity. Older adults, facing greater health risks, benefit more from reduced information barriers and expanded intervention channels. Those with lower education levels may lack access to traditional health information but gain more from the accessibility of digital tools. In contrast, highly educated individuals often already possess sufficient health resources, limiting the marginal utility of digital infrastructure. Similarly, middle-aged adults generally exhibit lower health service demand, leading to less pronounced effects. These results highlight the differentiated impacts of infrastructure policies across social groups and support a more equitable approach to digital governance and health equity.

Fourth, digital infrastructure development contributes to reducing health inequality among middle-aged and older adults. Existing literature has identified various drivers of health inequality, including climate change [49], racial discrimination [50], and unequal distribution of healthcare resources [51]. However, most studies focus on the exacerbating mechanisms of inequality, overlooking the potential of digital infrastructure to serve as a compensatory policy tool that improves health service provision and bridges information gaps. This study finds that digital infrastructure disproportionately benefits individuals with poorer health, suggesting a potential ‘compensatory effect’ that helps narrow health disparities. Mechanisms may include improved access to healthcare services, reduced barriers to health information among disadvantaged populations, and greater self-management capabilities supported by digital devices. Unlike prior research that primarily investigates the origins of health inequality, this study highlights the structural function of digital infrastructure in promoting health equity, highlighting potential considerations for the design of digital health policies.

This study, based on a quasi-natural experiment of the Broadband China pilot policy, makes the following contributions: First, it advances the literature by shifting attention from individual internet use to macro-level digital infrastructure as a public resource, demonstrating its spillover health effects in a rapidly digitalizing context like China. Second, it reveals the mediating role of health behaviors in the link between infrastructure and health. By enhancing information access, encouraging health-related consumption, and improving social interactions, digital infrastructure supports better health outcomes and enriches the theoretical framework of digital health governance. Third, it conducts a comprehensive analysis of health inequality and heterogeneity, demonstrating that digital infrastructure development can reduce intergroup disparities and generate compensatory benefits for disadvantaged populations. These insights contribute to empirical evidence that may inform precision health interventions and digital public service strategies.

Despite the comprehensive empirical evidence, this study has several limitations. First, the CHARLS data, while authoritative and nationally representative, are only available up to 2020. Given the rapid expansion of digital infrastructure since then, future research with updated datasets may reveal stronger or different health effects over time [52]. Second, the observed health improvements are statistically significant but modest in magnitude. Even though the changes are incremental, they reflect measurable benefits, which may accumulate over time and still hold practical relevance for policy and individual well-being. Third, despite employing city and year-fixed effects and a PSM-DID design, the non-random selection of pilot cities may leave residual bias. Differences in local resources, institutional capacity, or pre-existing health trends could partially confound the estimates, somewhat limiting the strength of causal inference. Finally, the mediating effects of physical exercise and health-related expenditures are statistically significant but small, and the causal pathway cannot be fully isolated; other unobserved factors or concurrent social policies may also influence these behaviors. Nevertheless, these findings provide partial evidence of the mechanisms through which digital infrastructure contributes to health improvements.

## Conclusion

Drawing on panel data from CHARLS from 2011 to 2020, this study leverages the quasi-natural experiment of the Broadband China pilot initiative to examine the impact of digital infrastructure development on the physical health and health inequality of middle-aged and older adults. It further investigates the mediating role of health behaviors and explores heterogeneity across population subgroups. The main results are as follows:

First, digital infrastructure development contributes to measurable improvements in the physical health of middle-aged and older adults, including reductions in limitations in activities of daily living. While these improvements are modest, they reflect incremental yet meaningful benefits at the population level, particularly given the macro-scale nature of the intervention. Unlike previous research focusing on individual internet use, this study highlights broader health effects arising from infrastructure-level initiatives.

Second, health behaviors serve as partial pathways through which digital infrastructure affects health outcomes. Specifically, infrastructure development is associated with increased physical exercise and more informed health-related expenditures, indicating that behavioral changes contribute to observed improvements.

Third, the health effects of digital infrastructure appear heterogeneous. Older adults and those with lower education levels tend to benefit more, while middle-aged adults and individuals with higher education show relatively limited improvements. These findings underscore the importance of addressing the digital divide in health and suggest that tailored interventions could be considered for different subgroups.

Fourth, digital infrastructure development may reduce health inequalities. Improvements are more pronounced among individuals with poorer baseline health, suggesting a compensatory effect that contributes to narrowing disparities in physical health outcomes.

Based on these findings, the study offers the following policy implications:

First, accelerating digital infrastructure development could enhance potential health spillover effects. Governments should continue expanding fiber-optic broadband and 5 G network coverage, strengthen smart healthcare and telehealth systems, and increase infrastructure investment to support more equitable health outcomes.

Second, empowering middle-aged and older adults through digital health tools may help optimize health behaviors. This can be achieved by offering subsidies and training to improve digital health literacy, promoting the adoption of wearable devices, health management applications, and online health education platforms to encourage active engagement in personal health management.

Third, precision-informed health policies tailored to subgroup differences are recommended. For older adults and individuals with lower education levels, policies could focus on enhancing digital health literacy and service accessibility, promoting age-friendly smart devices, and building online health support networks. For urban residents and more educated groups, advanced health data management and intelligent health interventions may further improve management efficiency.

Fourth, digital technology could be leveraged to reduce health inequality. Expansion of public digital health services and better integration of health insurance with digital platforms could help ensure that disadvantaged groups have access to affordable telemedicine and consultations. Simultaneously, strengthening data security and privacy protections is essential to prevent uneven distribution of digital health resources from exacerbating existing disparities.

## Data Availability

The data that support the findings of this study are available from the corresponding author, X.H., upon reasonable request.
